# Xenotransplantation: lessons from history, genetic engineering, and early clinical experience—an interview with Dr. David K. C. Cooper

**DOI:** 10.3389/frtra.2026.1827646

**Published:** 2026-05-08

**Authors:** Reza Abdi

**Affiliations:** Kidney Transplant Immunobiology Program, Division of Nephrology, David Geffen School of Medicine at UCLA, University of California, Los Angeles, CA, United States

**Keywords:** future perspectives, genetic-engineering, immunosuppressive therapy, industry, pigs, pioneers, xenotransplantation

## Abstract

Xenotransplantation has evolved from speculative experimentation into a scientifically grounded and increasingly applied clinical discipline, propelled by advances in immunology, molecular genetics, and genome engineering. In this interview, Dr. David Cooper, a pioneer in the field, reflects on the historical origins of xenotransplantation and traces key mechanistic breakthroughs, including the identification of carbohydrate xenoantigens, complement incompatibility, and innate immune barriers. Major milestones involving the organ-source pig include the development of αGal-knockout pigs, the expression of human complement-regulatory proteins, and the emergence of highly multi-gene–edited pigs enabled by CRISPR-based technology. Attention is given to kidney-specific challenges, including metabolic and physiologic incompatibilities, coagulation dysregulation, and the continued need for significant immunosuppressive therapy based on blockade of the CD40/CD154 co-stimulation pathway. Differing donor-engineering strategies adopted by various companies, unresolved barriers, such as swine leukocyte antigen (SLA) incompatibility, and lessons from early clinical experience are explored. Together, these insights highlight both the remarkable progress and the substantial challenges that remain before xenotransplantation can become a durable and scalable clinical solution.

## 1 Historical background: from early attempts to mechanistic understanding

### 1.1 Question: do you agree that xenotransplantation has occupied the imagination of humankind longer than almost any other medical intervention?

#### 1.1.1 Response

The fantasy of humans incorporating tissues from various animal species predates modern medicine and immunology as far back as mythology (1). Early clinical efforts during the mid-20th century in Europe and North America included nonhuman primate (NHP)-to-human kidney, heart, and liver transplantation (1). Even earlier, and perhaps more troubling, were non-scientific attempts, such as testicular xenografting, some promoted by charlatans or clinics claiming rejuvenation and enhanced vitality. The early clinical experiments by surgeons (such as Keith Reemtsma, James Hardy, and Leonard Bailey) were usually short-lived and were characterized by a poorly defined ‘rejection’ process or infectious complications (2).

Later, when pigs were used as organ donors, it became clear that xenograft failure was qualitatively distinct from allograft cellular rejection, driven by pre-existing immune mechanisms primed against animal tissues. These failures, while dramatic, were formative. They redirected the field from empiricism and toward mechanistic investigation. Understanding why xenografts failed became more important than simply intensifying immunosuppressive therapy, thereby laying the intellectual foundation for modern xenotransplant immunology. A search was initiated to identify the pig xenoantigens to which human antibodies bound, and to investigate the role of complement activation (3).

## 2 Personal motivation and key contributors to the field

### 2.1 Question: what drew you personally to xenotransplantation, and who were the other major figures shaping the field?

#### 2.1.1 Response

When I became involved in cardiac allotransplantation in the UK (initially as a researcher in the late 1960s and subsequently as a clinician in the late 1970s), it soon became clear to me that a major problem was the shortage of suitable human donor organs. At that time, patients might wait in an intensive care unit for weeks or months for a suitable heart, but then sadly die before one became available. Despite frequent efforts by the transplant community to educate the public about the need for organ donation, I realized then that there would never be sufficient organs to meet the demand unless we could identify an alternative animal source. In the early 1980s, when I was on the faculty at the University of Cape Town (where NHPs were readily available), working under the leadership of Professor Christiaan Barnard, who, as you know, carried out the world's first heart allotransplantation in 1967, I explored the possibility of using NHPs as a source, but clearly, these did not meet many of the necessary criteria. I therefore shifted my attention to the pig (4).

**Figure 1:**
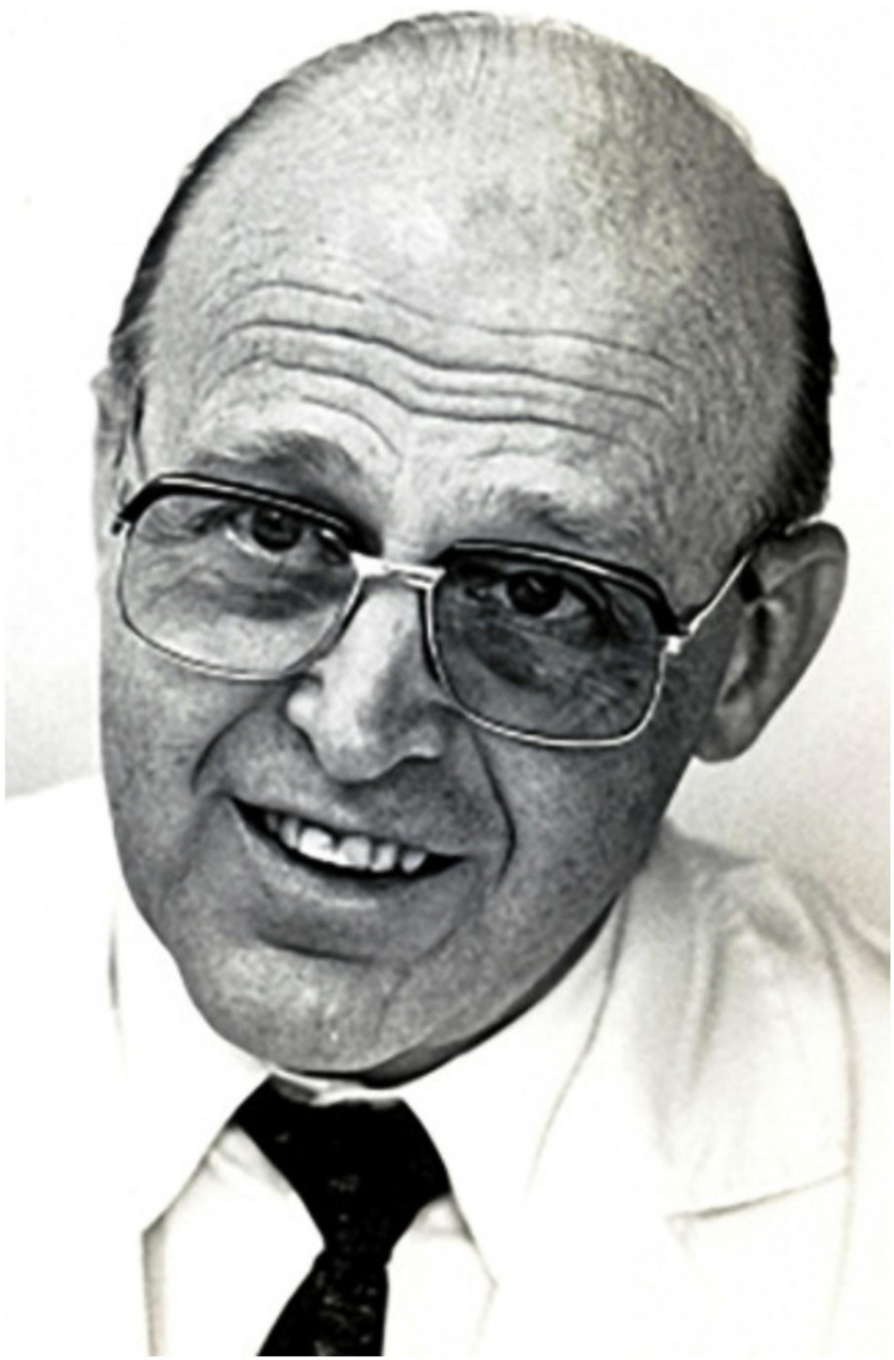
Guy alexandre (1934–2024).

**Figure 2:**
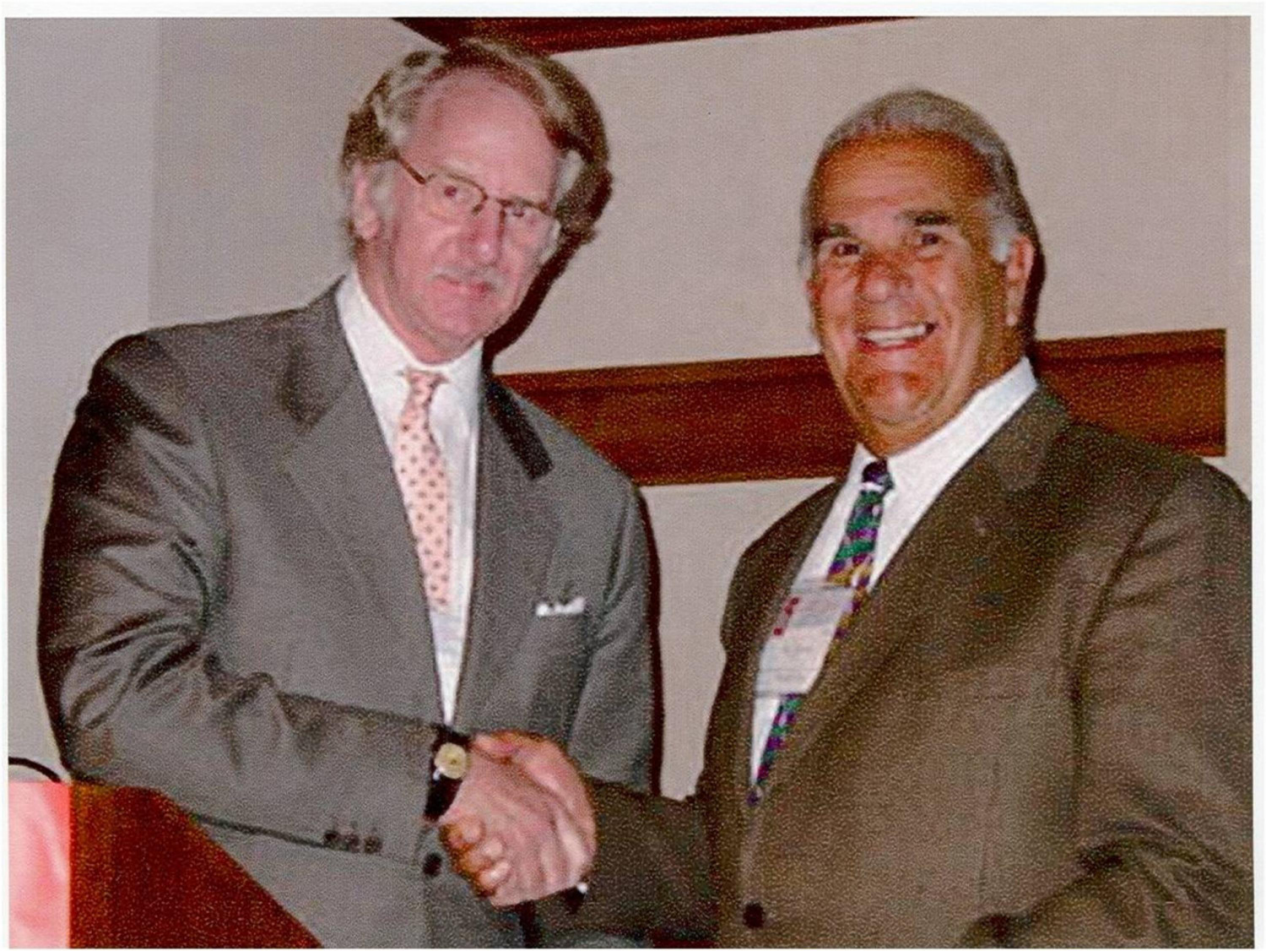
John Najarian (1927–2020) (right) receiving honorary membership of the international xenotransplantation association (IXA) from david cooper (then president) in 2001.

The late Belgian surgeon, Guy Alexandre (1934–2024) (Figure 1), whose contributions to allotransplantation included the first use of kidneys from human brain-dead donors and the first efforts to electively transplant across the ABO blood group barrier (for which I do not believe he ever received sufficient recognition), explored pig kidney xenotransplantation at about the same time (5). The extensive work in the 1960s and 1970s in the pig-to-dog model by John Najarian's group (Figure 2) provided some useful background for us (6).

**Figure 3:**
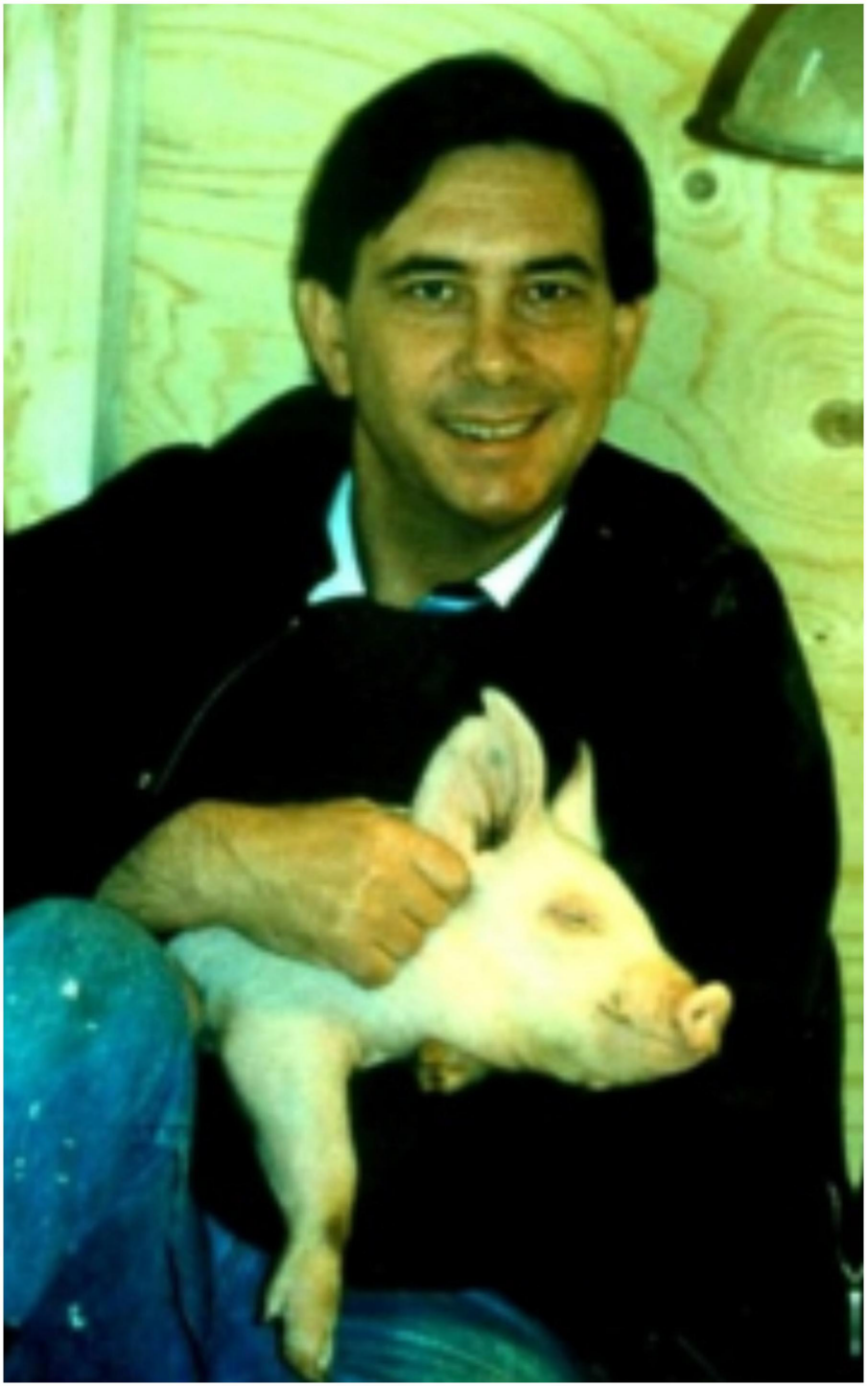
David White (1946–2017) with “astrid”, the first genetically-modified pig specifically produced for experimental xenotransplantation.

**Figure 4:**
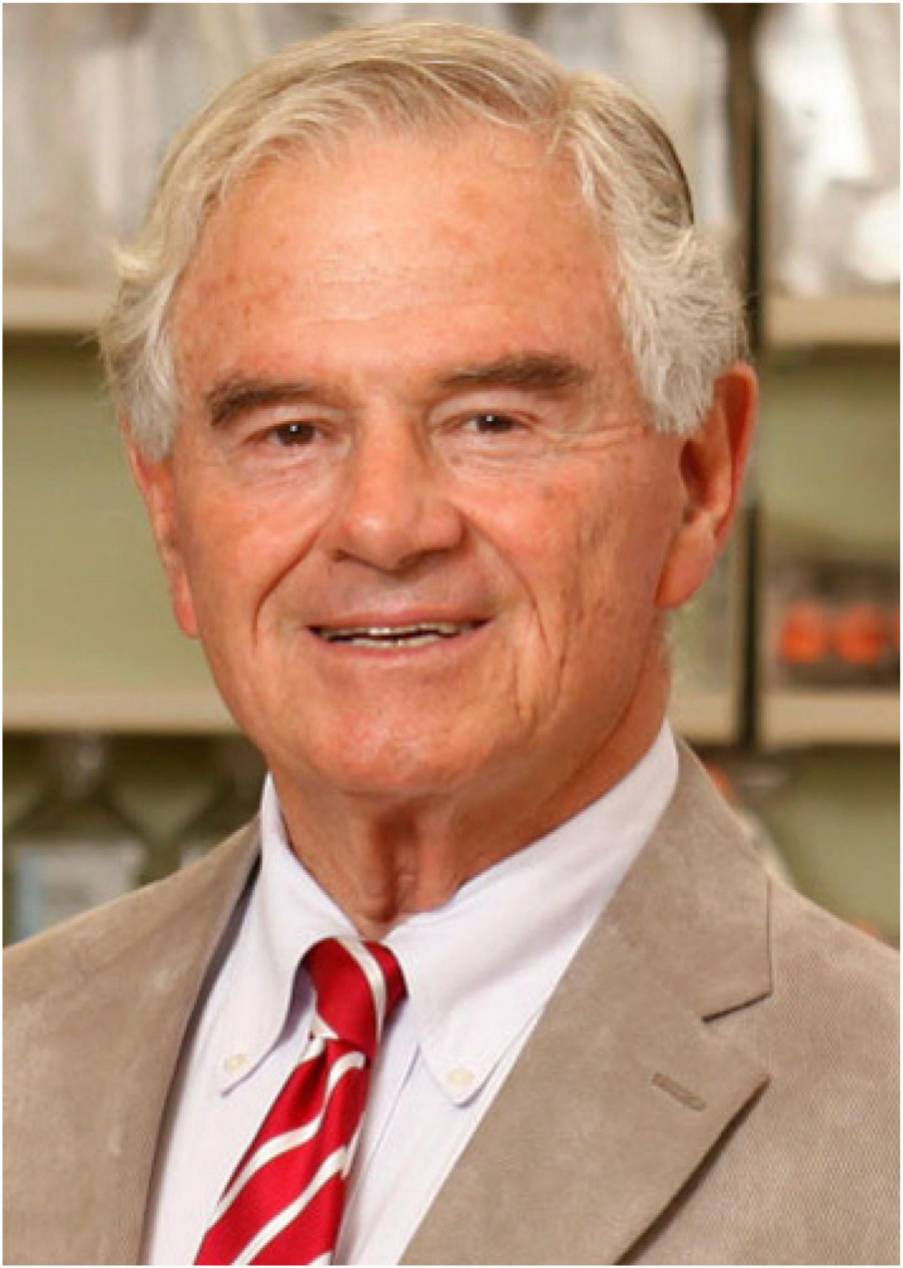
David Sachs.

One scientist who contributed significantly in those early days was my friend David White (1946–2017) (Figure 3) at the University of Cambridge in the UK, who was the first to produce a genetically engineered pig [expressing the human complement-regulatory protein [CRP] CD55 [decay accelerating factor]] that extended graft survival in the pig-to-NHP model from minutes or hours to days or weeks. Other visionaries at that time included Jeff Platt and Simon Robson in the USA, and three Australians—Tony d'Apice and Ian McKenzie, both of whom had spent time at Harvard-associated hospitals in Boston, and Mauro Sandrin. Both d'Apice and McKenzie were early presidents of the International Xenotransplantation Association (IXA). I must also not forget my very influential and greatly valued early collaborators, Eugene Koren (in Oklahoma City) and Raphael Oriol (in Paris). The field benefited from the contributions of investigators such as David Sachs (Figure 4), whose ambition was largely in tolerance induction (7), which proved difficult even in models of allotransplantation and, although progress has been made, has not yet proved successful in xenotransplantation.

**Figure 5:**
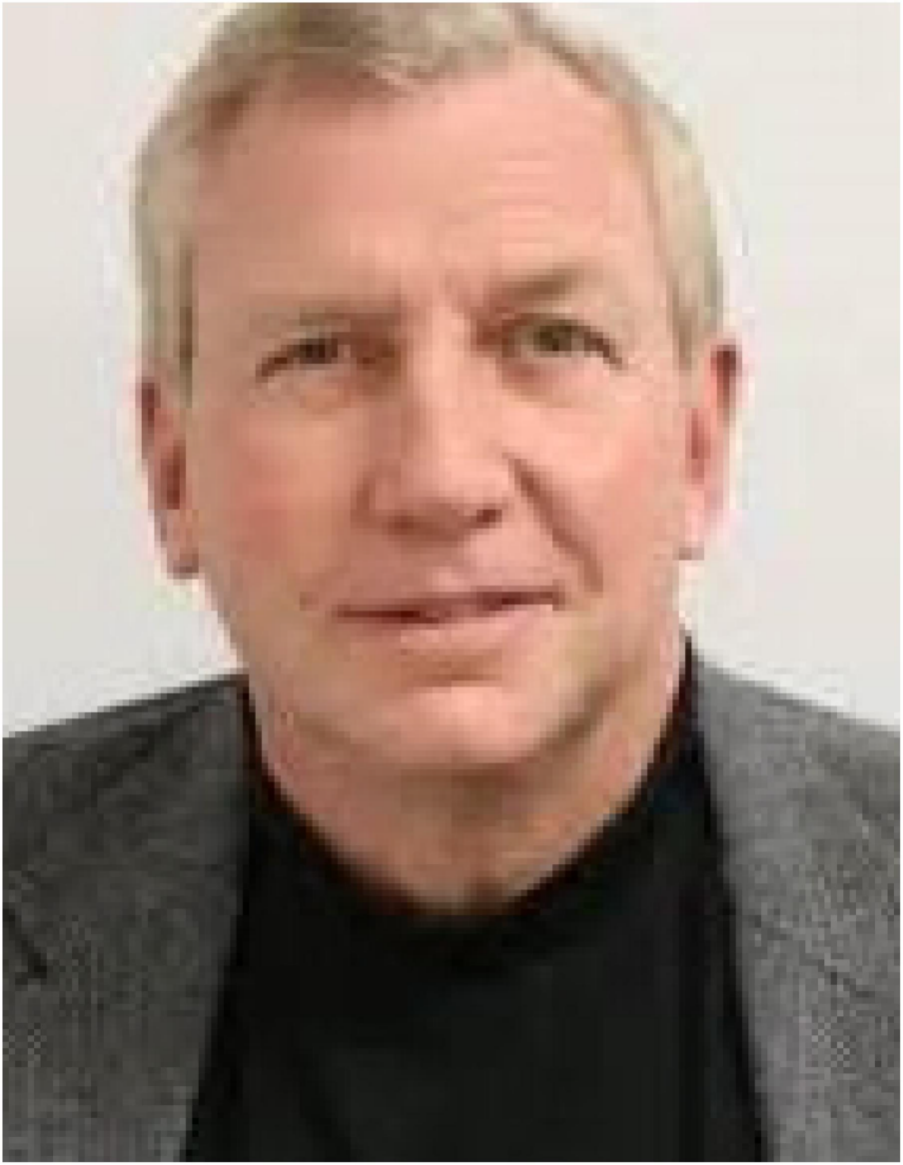
David Ayares (CEO and CSO, revivicor).

Xenotransplantation began to attract investigators who were willing to confront fundamental immunobiologic questions that could not be answered within the framework of allotransplantation. Once the key problems had been identified, a new generation of molecular geneticists collectively transformed xenotransplantation into a hypothesis-driven discipline grounded in immunology. After David White and his colleagues at Imutran in the UK, the field of genetic engineering was largely led by David Ayares (Figure 5) at Revivicor in the USA; without his persistence, progress would have been even slower than it was.

## 3 Key milestones in xenotransplantation

### 3.1 Question: what were the key milestones in xenotransplantation over the decades?

#### 3.1 Response

There were several milestones (Table 1), but they can largely be grouped under (i) genetic engineering of the organ-source pig and (ii) the introduction of CD40/CD154 T cell co-stimulation blockade as an immunosuppressive therapy.

**Table 1:** Selected major advances in xenotransplantation research (largely in pig-to-primate models).

Major events
1960s/1970s: Studies in pig-to-dog and dog-to-pig models (Najarian) (6)
1968: First report of pig-to-baboon liver Tx (Calne) (38)
1986: First report of pig-to-baboon heart Tx (Lexer/Cooper) (4)
1989: First report of pig-to-baboon kidney Tx (Alexandre) (5)
1992: First genetically-engineered pig (expressing human CD55) (White) (8)
1991: Definitive identification of Gal as a xenoantigen and of its importance in xenoTx (Good/Cooper, soon confirmed by d'Apice and McKenzie/Sandrin) (9, 10)
1993: Proposal to produce GTKO pigs as sources of organs for xenoTx (Cooper/Koren/Oriol) (12)
1996: First cloned large mammal, Dolly the sheep (Campbell/Wilmut) (39)
1996: Identification of Neu5Gc as a xenoantigen (Bouhours, and later confirmed by Zhu/Hurst) (17, 43)
1998: Confirmation of coagulation dysfunction after pig-to-NHP organ Tx (Ierino/Kozlowski/Robson) (40)
2000: First cloned pig (Polejaeva/Ayares/Campbell) (41)
2000: Report of failure of the calcineurin inhibitor, cyclosporine, to prevent sensitization to wild-type pig cells in primates and of the success of an anti-CD154mAb to prevent sensitization (Buhler/Cooper) (28)
2003/4: First GTKO pigs (Dai/Phelps/Ayares, and Lai/Kolber-Simonds/Hawley) (13, 14)
2005: First transplants of GTKO hearts (Kuwaki/Cooper) and kidneys (Yamada/Sachs) into NHPs (15, 16)
2014: Identification of Sda as a xenoantigen (Byrne/McGregor) (18)
2015: Confirmation of the prolonged inflammatory response to a pig organ in a NHP (Ezzelarab/Cooper) (20)
2015: First triple-knockout (TKO) pigs (Estrada/Tector) (19)
2017: First PERV-KO pigs (Niu/Church/Yang) (26)
2017+: Increasing survival of NHPs with life-supporting gene-edited pig organ grafts (several groups).
2022: First clinical pig heart Tx (Griffith/Mohiuddin) (30)
2024: First clinical pig kidney Tx (Kawai/Riella) (32)
2026: Identification of nephrotic-range proteinuria and thrombotic microangiopathy after pig-to-baboon kidney Tx (Kinoshita/Cooper) (29)
2026: First clinical pig liver Tx (Zhang/Sun) (42)

Gal, galactose-α1,3-galactose; GTKO, α1,3-galactosyltransferase gene-knockout; Neu5Gc, N-glycolylneuraminic acid; NHP, nonhuman primate; PERV, porcine endogenous retrovirus; TKO, triple (carbohydrate xenoantigen)-knockout; Tx, transplantation.

### 3.2 Overcoming complement incompatibility

Complement incompatibility emerged early as a central barrier. In kidney xenografts, this problem may be magnified by the organ's dense, highly specialized microvasculature. Unchecked complement activation leads to endothelial activation, upregulation of tissue factor, leukocyte recruitment, platelet aggregation, and microangiopathic thrombosis, features that resemble thrombotic microangiopathy rather than classical cellular rejection.

It was partially resolved by the work of David White and the late Gus Dalmasso (in Minneapolis), who independently noted that complement regulatory proteins (CRPs, which protect humans and animals from autologous complement injury) were largely species-specific. To mitigate this, pigs were engineered to express human CRPs, including CD46 (MCP), CD55 (DAF), and/or CD59, significantly improving vascular integrity and graft survival (8). The expression in pigs of one or more human CRPs provided some protection from human complement-mediated injury. As mentioned above, this made a very significant difference to pig heart or kidney graft survival in NHPs. It is often overlooked that the *level* of CRP expression is important. A high level of expression of a single CRP may be more effective than low levels of expression of two or more CRPs.

Complement dysregulation remains a small barrier, at least in the perioperative period, that needs to be addressed and prevented. Recently, we have administered a short course of a complement inhibitor, e.g., C1, C3, or C5 inhibitor, to the recipient in the perioperative period to suppress systemic complement activation.

### 3.3 Identification of carbohydrate xenoantigens

The identification of hyperacute rejection as an antibody-mediated process driven by natural antibodies recognizing carbohydrate antigens on porcine endothelial cells, similar to ABO incompatibility, was an important milestone. Pig galactose-α1,3-galactose (αGal) was identified as the dominant xenoantigen (9, 10).

**Figure 6:**
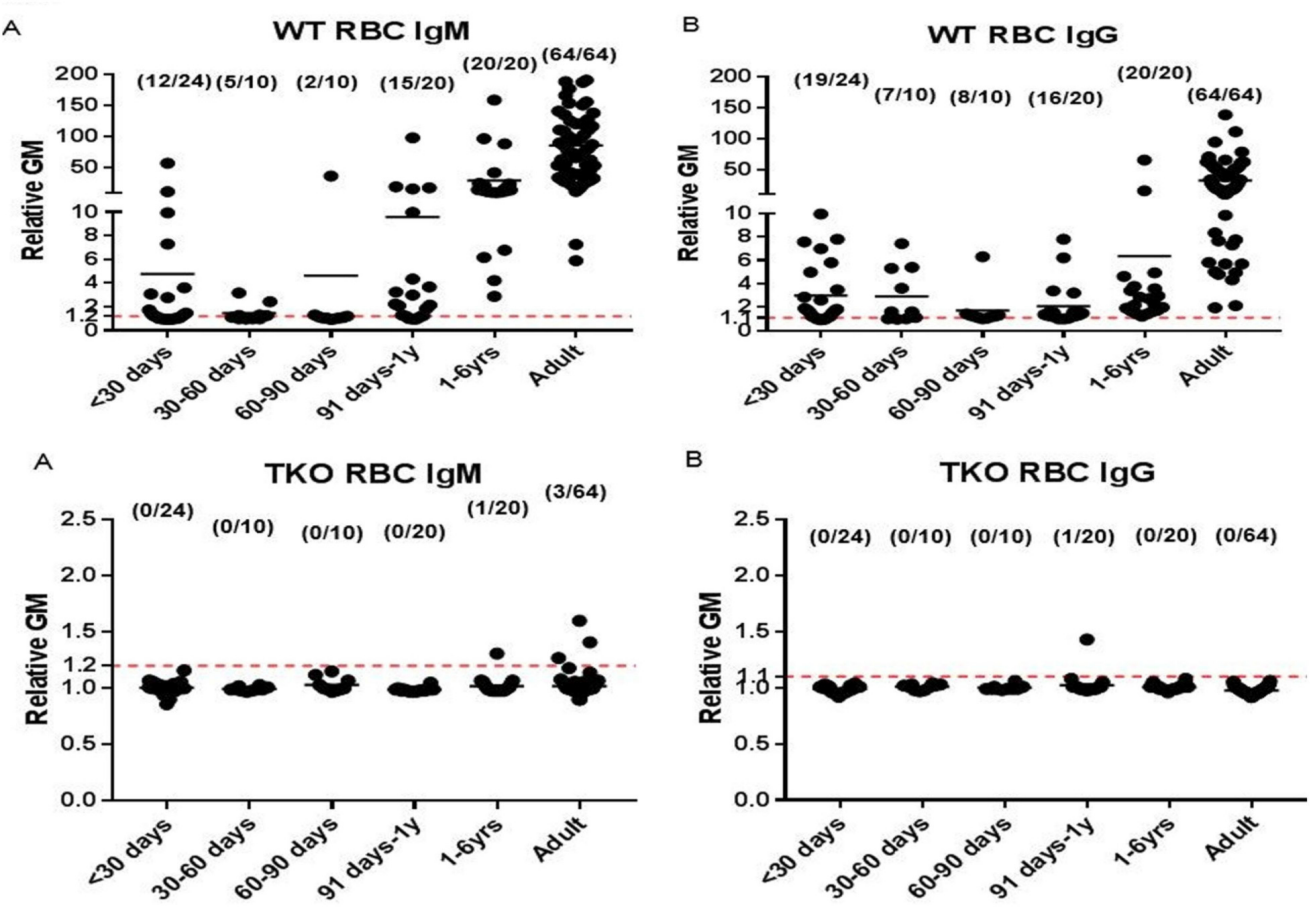
Geometric mean (GM) IgM and IgG binding and age correlation of human serum binding to wild-type (WT) (top) and triple-knockout (TKO) (bottom) pig red blood cells. (Note the difference in the scale on the *y* axis between the binding to WT and TKO cells.) [Reprinted with permission from (9)].

Anti-Gal antibodies are ubiquitous in humans and are believed to arise largely from chronic exposure to intestinal microorganisms that express Gal-like carbohydrate motifs. Hidetaka Hara and his colleagues demonstrated that anti-pig antibodies begin to develop during the first few months after birth and increase in titer until, and possibly throughout, adulthood (11) (Figure 6). Upon transplantation of a pig organ, these antibodies bind immediately to the graft endothelium, activate complement, and induce rapid injury, thrombosis, and graft loss.

The generation of α1,3-galactosyltransferase gene-knockout (GTKO) pigs [first advocated by my group in 1993 (12), but not technically possible until 2003, when they were produced by two companies (Revivicor and Immerge)] represented a conceptual breakthrough (13, 14). Survival of GTKO pig hearts and kidneys in NHPs increased to a maximum of 6 months (15, 16).

Together with David White's work, it demonstrated that genetic modification of the donor, rather than escalating immunosuppressive therapy in the recipient, could overcome a fundamental immunologic barrier. This decisively shifted the field toward donor-centered engineering strategies. A guiding principle of mine has been “the more you can do to the pig donor, the less you need to do to the human recipient.”

### 3.4 Beyond Gal-additional genetic engineering

While αGal-knockout was essential, it was not sufficient (15, 16). Additional pig carbohydrate antigens, including Neu5Gc (17) and Sda (18), also elicit antibody responses in humans (Table 2). Like anti-Gal antibodies, these responses are believed to be shaped by the human microbiome and dietary exposure and contribute to delayed antibody-mediated injury and chronic rejection. Deletion of expression of all three of the known carbohydrate xenoantigens (Gal, Neu5Gs, Sda), first achieved by Estrada and his colleagues in 2015, was another major step forward (19).

**Table 2:** Pig gene knockouts and representative human ‘protective’ transgenes introduced into the pigs.

Category	Gene/Transgene	Function
Pig gene knockouts (KO)	α1,3-galactosyltransferase (Gal)	Removes major xenoantigen (Gal)
	CMAH (Neu5Gc)	Removes xenoantigen (Neu5Gc)
	*β*4GalNT2 (Sda)	Removes xenoantigen (Sda)
	Porcine endogenous retrovirus (PERV)	Inactivation of viral elements (i.e., 59 copies)
Human transgenes	CD46 (MCP)	Complement regulatory proteins
	CD55 (DAF)	
	Thrombomodulin	Anticoagulant
	Endothelial protein C receptor	Anticoagulant
	Heme oxygenase-1 (HO-1),	Anti-inflammatory
	TNF-α induced protein 3 (A20)	Anti-inflammatory
	CD47	Inhibits phagocytosis

**Figure 7:**
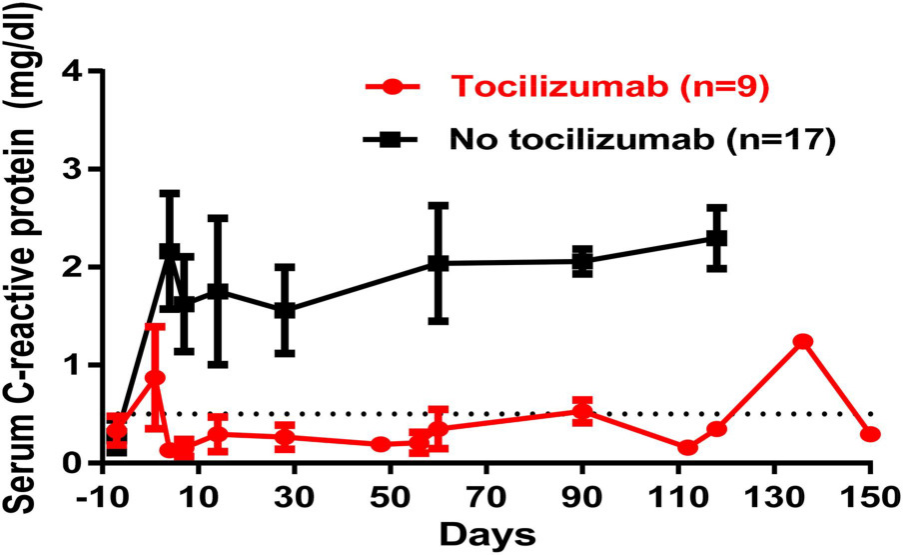
Serum C-reactive protein (C-RP) responses to gene-edited pig kidney or artery patch transplants in immunosuppressed baboons being treated with or without tocilizumab (anti-IL-6RmAb). [Reproduced with permission from (21)].

Early studies by Simon Robson and colleagues at Beth Israel Hospital in Boston and others drew attention to the inflammatory response to a xenograft (3), which Mohamed Ezzelarab later demonstrated in the pig-to-NHP model to be prolonged (21) (Figure 7). As inflammation can augment the immune response, both genetic engineering of the pig (22) (Table 2) and the administration of agents that suppress IL-6 production (23) (Table 3) have been incorporated into the regimen.

**Table 3:** Immunosuppressive regimen administered to recent baboons with life-supporting gene-edited pig heart grafts (with survival >24 months in one case). (Transplant = day 0).

Treatment protocols
*Induction*
Anti-thymocyte globulin (ATG) 2.5 mg/kg IV on days −3 and −1
Anti-CD20mAb (Rituximab) 10 mg/kg IV on day −2.
C1 esterase inhibitor 17.5 units/kg IV (Berinert) on days 0 and 2.
*Maintenance*
Anti-CD154 mAb (Tegoprubart) 20 mg/kg IV on days −2, −1, 0, 3, 7, 10, 14, and weekly to achieve a trough level of >1,000 µg/mL by day 0 and >500 µg/mL thereafter.
Rapamycin daily IM (from day −5) to maintain a 24 h trough level of 8–12 ng/mL.
Methylprednisolone 10 mg/kg IV, tapering to 0.25 mg/kg IM daily by day 7, and then daily.
Tocilizumab (IL-6R blockade) 8 mg/kg IV on days 0, 7 and 28, and then monthly

Current organ-source pigs therefore incorporate multiple carbohydrate knockouts (Table 2) (19), reducing the graft's antigenic footprint and limiting innate immune activation. In addition, they may express several human proteins that protect against complement- and coagulation-mediated dysfunction, inflammation, and other causes of immune injury (22) (Table 2). These findings underscore that xenotransplantation is not a single-barrier problem but a layered immunologic challenge.

### 3.5 Question: are we now experiencing the same sort of struggle seen in the early days of organ allotransplantation in Paris and Boston?

#### 3.5.1 Response

Not exactly. We are beginning clinical trials at a far more advanced stage than our predecessors were in the 1950s concerning allotransplantation. For example, René Küss, one of the French pioneers in the field, recounted to me how he and his colleague would wait in a Paris prison until a criminal was guillotined and his headless body dragged into the room. The surgeons, on their knees, would remove the kidneys. They had no concept of ischemic injury, no immunosuppressive agents, and no successful experience in animal models (2). Today, we are far ahead of the early French teams and the Peter Bent Brigham pioneers, thanks to the zealous work of scientists and physicians worldwide who have steadily improved transplant outcomes.

## 4 Genetic engineering as a defining milestone

### 4.1 Question: should advances in genetic engineering be considered a major milestone in their own right?

#### 4.1.1 Response

Absolutely. Genetic engineering of the organ-source pig has been essential to our progress. For several years, David Sachs and I tried to make progress before genetic engineering of the pig was readily available, e.g., by antibody depletion in the recipient, etc. (24), and basically got nowhere. Although several methods of genetic engineering were sequentially introduced (Table 4), the methods were laborious, time-consuming, and inefficient. So, we have to thank pioneers such as David Ayares and Yifan Dai at Revivicor and their counterparts at Imutran, Immerge, and Nextran (companies that sadly went out of business) for their perseverance.

**Table 4:** Timeline for application of evolving techniques for genetic engineering of pigs employed in xenotransplantation.

Year	Technique
1992	Microinjection of randomly-integrating transgenes
2000	Somatic cell nuclear transfer (SCNT)
2002	Homologous recombination
2011	Zinc finger nucleases (ZFNs)
2013	Transcription activator-like effector nucleases (TALENs)
2014	CRISPR/Cas9*

* CRISPR/Cas9, clustered randomly interspaced short palindromic repeats and the associated protein 9.

In more recent years, the introduction of CRISPR-based genome editing marked a decisive inflection point for xenotransplantation. It helped transform the field from a largely stepwise, single-gene effort into a scalable, systems-level engineering endeavor. CRISPR technology has made genetic engineering of pigs simpler, faster, and more cost-effective. Nevertheless, the remarkable foundational progress achieved by earlier investigators was extremely important and should not be underestimated or overlooked.

Using CRISPR technology, pigs have been engineered to simultaneously knock out multiple carbohydrate antigens, insert human complement and coagulation regulatory genes, express immune-modulatory molecules, such as CD47, to reduce macrophage-mediated phagocytosis, and inactivate porcine endogenous retroviruses (PERVs). Whereas in the early days the pigs were modified incrementally, the technology is now available to make multiple gene edits simultaneously.

## 5 Companies in xenotransplantation

### 5.1 Question: what are the main companies and strategic differences in xenotransplantation?

#### 5.1.1 Response

In the USA, only two companies are currently involved in clinical organ xenotransplantation (Revivicor and eGenesis), but at least three others are developing similar technologies. In China, one company is participating in clinical studies (ClonOrgan). They are all producing pigs with similar genetic modifications, although there are some differences between them.

For example, Revivicor has developed its pigs from large, domesticated breeds, e.g., Large White, whereas eGenesis and ClonOrgan pigs are derived from Yucatan and Bama miniature swine backgrounds, respectively. The observation that Large White pig organs grow rapidly in NHPs after transplantation has necessitated that Revivicor follow the Munich group's lead by knocking out the gene for growth hormone receptors to prevent the transplanted organs from growing too large, too quickly (25). This is particularly problematic when a pig heart is transplanted into the restricted confines of the chest. However, when pig islets are required, rapid growth in the source pig (and its pancreas) may be advantageous, as more islets will be procured.

Following the work of geneticist George Church, eGenesis has also inactivated the genes for porcine endogenous retroviruses (PERVs), thereby negating any risk from them (26). Revivicor takes a different approach to reducing the risk of PERV activation. However, there is currently little evidence that PERVs would be problematic.

Fortunately, there has usually been close collaboration between academics and the private sector. For example, Henk-Jan Schuurman was immensely supportive to researchers when he worked sequentially for Sandoz, Novartis, Imutran, Immerge, and finally as an independent consultant. Without the expertise and close collaboration of companies such as Revivicor/United Therapeutics and eGenesis, we would not have progressed to where we are today.

## 6 Swine leukocyte antigens (SLA)

### 6.1 Question: why has swine leukocyte antigen (SLA) incompatibility received less attention?

#### 6.1.1 Response

**Figure 8:**
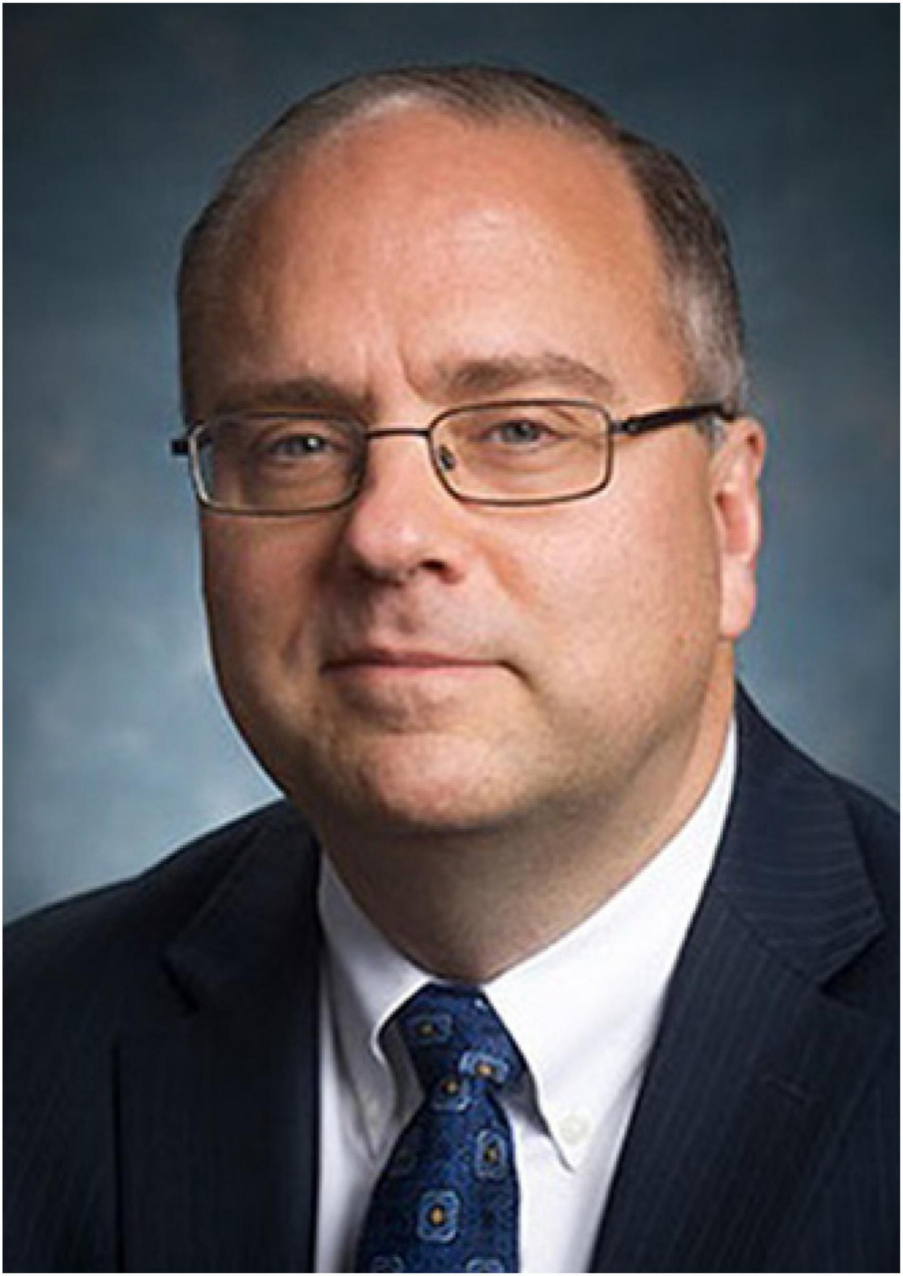
A.Joseph (Joe) Tector.

Unlike the three major carbohydrate antigens, swine leukocyte antigens (the pig equivalent of HLA) remain intact in current pigs and, while they probably continue to stimulate adaptive immune responses, play a less significant role in graft rejection than might be anticipated. However, Joe Tector (Figure 8) at the Makana company is investigating their role intensively (27). A complete SLA knockout has been of concern due to the perceived risk of rendering the pigs immunodeficient. We remain uncertain about the role of SLA mismatch in the outcome of a xenograft. However, the potent immunosuppressive regimens we use successfully suppress the adaptive immune response to SLA for a prolonged period. It is likely, however, that SLA may play a significant role in the development of chronic rejection.

## 7 Exogenous immunosuppressive therapy

### 7.1 Question: is the level of immunosuppression currently required sustainable?

#### 7.1.1 Response

I believe that it is. Having been involved in clinical organ allotransplantation as early as the late 1970s, the intensity of the immunosuppressive therapy we administer to the NHP or human recipient of a xenograft today (Table 3) is little different from what we administered for many years to the allograft recipient. I believe this opinion is supported by the relatively low incidence of infectious complications in experimental animals and in patients who have received a pig xenograft in recent years. Although the regimens exceed those used in today's allotransplantation, I believe that, with clinical experience and improved gene editing, the level of immunosuppressive therapy can be gradually reduced.

One important factor is our use of agents that block the CD40/CD154T cell co-stimulation pathway [first introduced into xenotransplantation by Leo Buhler in 2000 (28)]. I believe these remarkable agents (produced by companies such as Eledon and Tonix) will soon become routine in both clinical allotransplantation and xenotransplantation. In NHP *allotransplantation* experiments by my colleagues at MGH (Richard Pierson and Tatsuo Kawai), a single injection of an anti-CD154 mAb, administered weekly or every 2 weeks (with *no* induction therapy and *no* additional maintenance therapy), prevents rejection. Furthermore, these agents do not have the toxicity of the calcineurin inhibitors, and there have been very few complications.

Although this minimalist regimen is insufficient to protect a xenograft (29), the full anti-CD154mAb-based regimen is *not* excessive (Table 3). When my group was based at the University of Pittsburgh, we combined CD40/CD154 co-stimulation blockade with rapamycin, which has proved largely successful (29), though others have found mycophenolate mofetil or tacrolimus to be comparably effective.

With further additional gene edits aimed at controlling the adaptive immune response (of which there are several possibilities), there is every reason to believe that exogenous therapy can be steadily reduced. Indeed, I believe that eventually, *no* exogenous therapy will be required-protection will be achieved entirely through gene edits in the organ-source pig. Until then, we will need ongoing guidance from experts in transplant infectious diseases, such as Jay Fishman and Joachim Denner, who have contributed significantly to our progress.

## 8 Support from NIH vs. private sector

### 8.1 Question: would we have progressed to where we are today without the support of the private sector?

#### 8.1.1 Response

Definitely not. Although both the National Institutes of Health (NIH) and industry support have been essential, the costs of genetically engineered pigs have been borne primarily by the companies that provided the pigs, i.e., originally by Imutran, Immerge, and Nextran, and more recently by Revivicor/United Therapeutics, eGenesis, and Makana. Although NIH grants largely funded the pig-to-NHP experiments, the companies covered the immense costs of producing and housing the pigs. Martine Rothblatt of United Therapeutics has been especially supportive of xenotransplantation.

Given the enormous investment the private sector has already made in xenotransplantation, I would suggest that further support will only be sustained if there is continued progress that promises a successful and profitable future. However, I am very optimistic that such progress will be forthcoming.

## 9 Early clinical experience and regulatory landscape

### 9.1 Question: what have we learned from our early clinical experience?

#### 9.1.1 Response

Pig organ transplantation into deceased human donors did not provide any essential new information, but this experience (i) confirmed that hyperacute rejection did not occur (something we had known for years from extensive *in vitro* pig-to-human studies and *in vivo* studies in the pig-to-NHP model), and, importantly, (ii) drew the attention of the public and the US Food and Drug Administration (FDA) to the progress that had been made in xenotransplantation research.

The two heart xenotransplants at the University of Maryland revealed (i) the great importance of selection of patients with a realistic chance of recovering from severe and prolonged debility, (ii) the difficulty of managing immunosuppressive therapy in such patients, (iii) the need to avoid administration of medical products that potentially contain anti-pig antibodies, and (iv) the need for more sensitive tests to ensure the absence of pathogenic microorganisms in the pig organ graft (30, 31).

At the MGH (32), two of the four pig kidney transplants carried out to date (and the one in China) have underscored that our present ability towards immunologic control may be insufficient, as proteinuria and/or thrombotic microangiopathy have occurred in the absence of classical features of rejection or of measurable increases in anti-pig antibody (Leo Riella, personal communication 2025; Ke-Feng Dou personal communication 2025). This suggests that further gene editing may need to be targeted at coagulation dysregulation and podocyte injury (33). Early innate-type injury to the graft remains a key barrier and may be a greater hurdle than any remaining adaptive immune response.

If the immune response is controlled, gene-edited pig hearts appear to function well in NHP recipients. After clinical pig kidney transplantation, physiologic integration and metabolic compatibility have been explored, confirming observations from the experimental laboratory indicating that pig renin is not functional in primate recipients; it also appears that patients may require recombinant human erythropoietin support. In the future, these deficiencies may be resolved by judicious gene editing.

These experiences have informed ongoing discussions with the FDA, which currently permits pig kidney xenotransplantation under ‘compassionate-use’ extended criteria and has approved two formal clinical trials, emphasizing safety and rigorous monitoring.

## 10 Concluding perspective

### 10.1 Question: where do you see the field going from here?

#### 10.1.1 Response

Clinical trials of pig kidney and heart xenotransplantation will continue cautiously, with those involving the kidney seeking solutions to the thrombotic microangiopathy and proteinuria that we are experiencing today. Heart xenotransplantation will initially be aimed at bridging infants with complex congenital heart disease until a suitable allograft becomes available (34). In this respect, almost all available evidence indicates that immune sensitization to pig antigens will *not* be detrimental to a subsequently transplanted allograft (35).

In patients with fulminant liver failure, pig livers will be utilized for *ex vivo* perfusion as a bridge to either recovery of the native liver or until a suitable allograft becomes available. However, we will require improved gene-edited pigs, particularly to prevent coagulation dysregulation, to justify the permanent replacement of a human liver with a pig organ. Similarly, progress in pig lung xenotransplantation, investigated for many years by Robin Pierson and Lars Burdorf, is proving stubbornly slow despite multiple gene edits and multiple drug therapies. In view of the major public health problem of Type 1 diabetes, pig islet xenotransplantation, spearheaded in the early days independently by Bernhard Hering and Rita Bottino, is a field with immense potential (36, 37).

Xenotransplantation has reached a stage where failures are informative rather than discouraging. Continued progress will depend on integrating immunology, genetic engineering, physiology, and clinical research. Advances are likely to be incremental rather than revolutionary. However, when one looks back on the immense impact of a single novel drug, such as azathioprine (introduced by Calne and Murray) or of cyclosporine (introduced by Calne, Borel, and White) in allotransplantation, a ‘revolutionary’ breakthrough in xenotransplantation remains quite possible.

I confidently predict that eventually xenotransplantation will relegate allotransplantation to a topic of historic interest only.

## Data Availability

The original contributions presented in the study are included in the article/Supplementary Material, further inquiries can be directed to the corresponding author.
